# Chemoselective and stereoselective charge transfer dynamic quenching with triazacyclononane europium and terbium tetra-azatriphenylene complexes

**DOI:** 10.1039/d5dt03023d

**Published:** 2026-03-17

**Authors:** Xinyi Wen, Dominic J. Black, Robert Pal, David Parker

**Affiliations:** a Department of Chemistry, Hong Kong Baptist University Kowloon Tong 999077 Hong Kong China davidparker@hkbu.edu.hk; b Department of Chemistry, Durham University South Road Durham DH1 3LE UK

## Abstract

Dynamic quenching of the terbium and europium excited states of chiral 9-coordinate complexes incorporating a tetra-azatriphylene sensitising group is shown by emission and CPL spectroscopy to be both chemoselective and stereoselective for electron rich phenols that include (*S*)-DOPA, dopamine, homovanillic acid and (*S*) and (*R*)-Trolox; the efficiency of dynamic quenching is determined neither by the charge nor the oxidation potential of the quenching species.

## Abstract 2

Dynamic quenching of the lanthanide excited state is greatest for Tb complexes compared to Eu and reveals selective quenching of the *Δ* enantiomer by *R*-Trolox.

## Graphical abstract

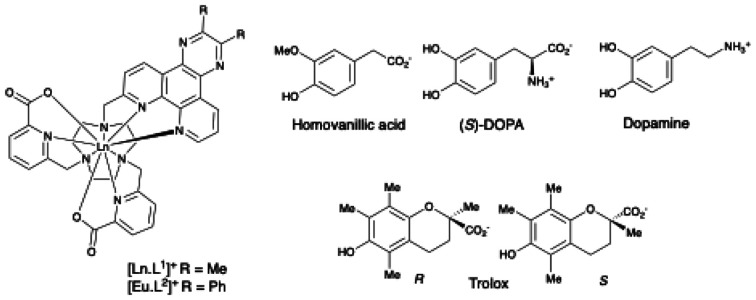

## Introduction

We report cases of chemoselectivity and stereoselectivity in excited state quenching of the long-lived excited states of luminescent terbium and europium complexes, that are neither driven by electrostatic attraction nor the redox potential of the quenching species.

Complexes of the rare earth ions are prized for their large pseudo-Stokes’ shifts, sharp emission lines and long radiative lifetimes, but their photoluminescence efficiency can be severely compromised by competing electron or charge transfer processes.^1,2^ There are several processes that can reduce the overall lanthanide photoluminescence quantum yield: charge transfer interference from low lying LMCT or MLCT states may overlap energetically with the excited lanthanide levels providing a radiationless deactivation pathway that can be a particular problem at elevated temperatures particularly for inorganic hosts or hybrid materials;^3,4^ photoinduced electron transfer (PET) depletes intermediate ligand-based singlet, ICT or triplet excited states for ligands with redox-active groups that can donate or accept electrons, competing with the rate of energy transfer to the lanthanide, thereby reducing emission efficiency;^5,6^ the structural rigidity and degree of shielding from solvent of the excited rare earth ion, often involving vibrational energy transfer is also a major problem with more conformationally mobile systems.^7,8^ Finally, electronic energy transfer from the lanthanide excited state itself, either intermolecularly to a matched acceptor, *e.g.* in a temperature independent FRET process,^9,10^ or intramolecularly in thermally activated processes that repopulate higher vibrational levels of the sensitising chromophore triplet or ICT state, highlighted in recent work.^11,12^

Most commonly, the strategies that have been devised to optimise overall photoluminescence efficiency in solution are based on the use of more rigid, non-redox active ligands to suppress PET, in which the sensitising chromophore triplet or ICT excited state is energy-matched to the given lanthanide excited state manifold.^13–15^ In addition, the use of ligand systems is required that effectively shield the excited state ion from vibrational and solvent quenching, *e.g.* by OH, NH, CH, C=O and aryl ring vibrations, among others.^7,8^

Having understood these basic issues, the design of responsive emissive probes based on lanthanide luminescence has attracted much recent attention,^16–18^ and can be signalled by modulation of emission intensity, lifetime or circular polarisation (CPL).^19–21^ Amongst these, ratiometric analysis based on differential quenching of Eu/Tb emission in complexes of a common ligand has figured prominently.^22^ Similarly, reversible displacement of bound or proximate solvent molecules in the presence of a target analyte or quenching molecule has been used with chemoselectivity profiles (*e.g.* for citrate, lactate and hydrogencarbonate) created by ligand structural design and the intuitive use of charge/steric complementarity.^23,24^ Differential quenching of the metal excited state (*e.g.* vibrational quenching by bound waters, Tb > Eu) also offers a strategy for devising selective probes, and has been used in the analysis of electron rich species, such as urate and ascorbate, based on the greater sensitivity to quenching of the ^5^D_4_ Tb excited state over the lower lying ^5^D_0_ Eu state.^24–26^

With this background in mind, we have been examining the quenching sensitivity of well-defined chiral complexes of Eu/Tb incorporating new electron poor tetra-azatriphenylene (or dipyridoquinoxaline, ‘dpq’) sensitisers. In particular, we were encouraged to examine the behaviour of new complexes, based on the structurally rigid triazacyclononane ligand framework, using nonadentate ligands some of which have been successfully resolved using chiral HPLC, and assess the sensitivity of these recently defined systems to excited state quenching by electron rich species, paying attention to the impact of complex charge and temperature on quenching efficiency.^27–29^

## Results and discussion

The lanthanide excited state can be quenched in several different ways that include electronic energy transfer (*e.g.* FRET), charge or electron transfer and vibrational energy transfer to matched proximate *infra*-red oscillators. The chiral drug, l-DOPA, and its important metabolites, dopamine and homovanillic acid (HVA) were selected for study, as examples of biologically relevant phenols, in order to test the hypothesis that the sensitivity of the chiral cationic complexes [LnL^1,2^]^+^ to excited state quenching by these electron rich species should be determined by charge complementarity or quencher redox potential.

### Complex resolution and stability to enantiomer interchange

**Fig. 1:**
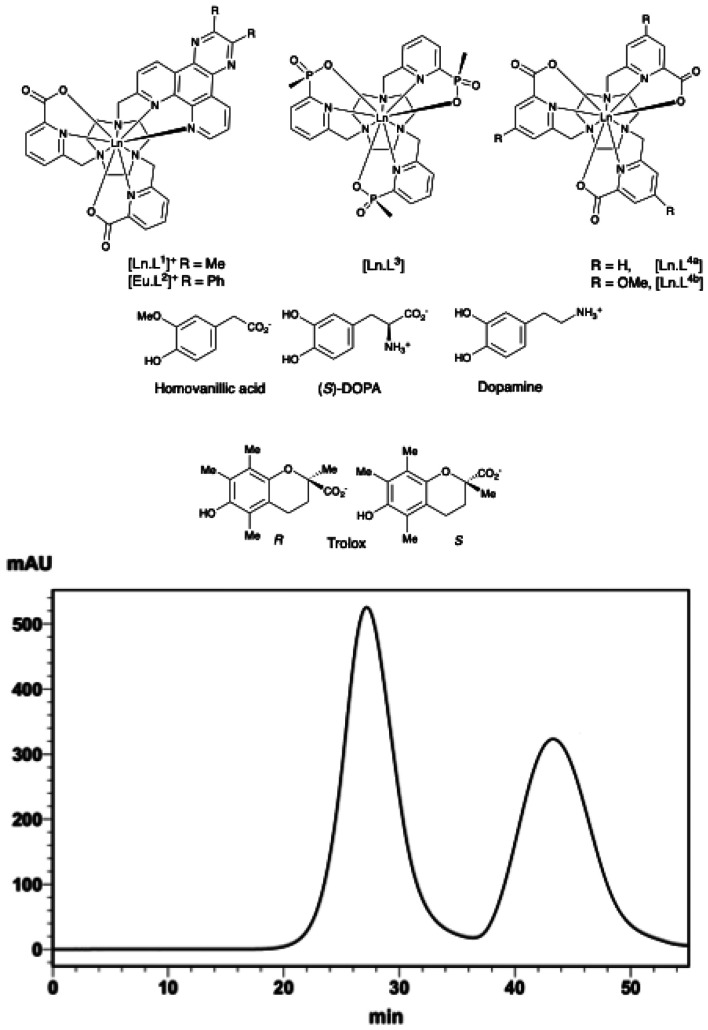
Resolution of [TbL^1^]^+^ using a CHIRLAPAK-1B HPLC column (eluant: EtOH/MeOH/TEA/TFA; v/v/v/v 50 : 50 : 0.5 : 0.3; 297 K).

The lanthanide(iii) complexes based on nonadentate 9-N_3_ (tacn) ligands, [LnL^1,2^]^+^, were prepared by minor adaptations to the recently reported methods (SI) and details of their characterisation have already been given in detail.^24,25^ The resolution of the chiral complexes [EuL^1^]^+^, [TbL^1^]^+^ and [EuL^2^]^+^ was examined using an analytical CHIRALPAK-IB N-5 250 × 4.6 mm 5 μm column, with an isocratic solvent system (EtOH/MeOH/TEA/TFA; v/v/v/v 50 : 50 : 0.5 : 0.3). The enantiomers of each complex separated successfully under these conditions, and the samples were isolated at room temperature (24 °C). The ratio of each enantiomer in the racemic mixture was 50/50, based on integrated area calculation, although they had different peak widths. For instance, the retention times of the isolated enantiomers for [TbL^1^]^+^ were 27.2 and 43.3 min, respectively (Fig. 1). Prior work with related chiral HPLC columns has shown that the *Δ* enantiomer of such helically chiral complexes based on 9-N_3_, *e.g.* [LnL^4a^] and [LnL^4b^] consistently eluted first,^27^ and the same assignment of absolute configuration was tentatively made at this point (*vide infra*).

The stability with respect to enantiomer interchange was investigated using chiral HPLC to assess enantiomeric purity variation, examining the change in the enantiomeric excess as a function of temperature and time. Forty percent of [TbL^1^]^+^ had racemised at 297 K after 48 h, and with [EuL^2^]^+^, 20% of the complex had racemised after 24 h storage at room temperature, notwithstanding storage as a glassy oil. The effects of temperature and light were hypothesised to determine the relative rates of [Ln^1,2^]^+^ enantiomer interchange. However, no significant difference in the rate of enantiomer exchange was found between light and dark conditions. Indeed, 29% enantiomer exchange had occurred after 24 h at 4 °C, and only 5% occurred after storage for 48 h at −25 °C, indicating that temperature is the expected primary factor governing rates of racemisation for these complexes in water.

These complexes undergo enantiomerisation (or enantiomer interchange) much more quickly in solution than analogous charge neutral 9-coordinate complexes based on triazacyclononane. For instance, the enantiomers of the tris-methylphosphinate complex, [EuL^3^], racemise only slowly, even when heated to 60 °C in H_2_O, with a half-life of 185 (±20) h.^27,28^ Similarly, the tris-carboxylate complex [EuL^4a^] racemised in water with a half-life of 240 h (±35) under the same conditions.

The occurrence of significant racemisation at ambient temperature in solution made it difficult to undertake accurate kinetic chiroptical studies in solution. However, using freshly separated samples maintained at dry ice temperatures whenever possible, the CPL spectra of [EuL^2^] (approximately 45% racemised samples) and [TbL^1^] (*ca*. 8% racemised samples) complexes were obtained, (Fig. S2) allowing a spectral correlation with the HPLC elution order. Thus, a *Δ* configurational assignment was given in each case to the first eluting enantiomer, in accord with earlier studies.^27–29^ The sample percentage enantiomer excess values noted above were estimated by chiral HPLC, immediately following the CPL spectral analysis.

### Luminescence quenching studies with dopamine, DOPA, homovanillic acid and Trolox

l-DOPA is a zwitterion at ambient pH, while dopamine is a mono-cation and HVA is a singly charged anion under these conditions and might be expected to promote collisional encounter because of a favourable, long range coulombic interaction. On the other hand, the one and two electron oxidations of the two catechols occur at a lower potential compared to HVA, and for each of these three examples, the reported oxidation potentials are pH dependent and show rather complex reversibility behaviour.^30,31^ In contrast, the water-soluble, chiral vitamin E analogue, Trolox, shows a relatively well-defined one electron oxidation at +0.48 V, which is a similar value to the single electron oxidation potential of many 1,2-catechols (+0.53 V *vs*. NHE, pH 7.4), that are charge neutral at ambient pH.^32–34^

**Fig. 2:**
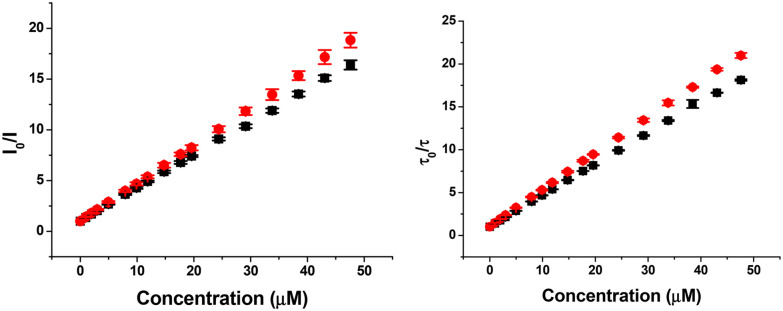
Variation of the [TbL^1^]^+^ emission intensity (left) or lifetime (right) *versus* Trolox concentration (*S*-black; *R*-red). Stern–Volmer quenching analysis shows the linear behaviour in emission intensity (*I*_0_ = intensity without quencher) and lifetime variations (*τ*_0_ = lifetime without quencher), (5 μM complex, pH 7.4, 0.1 M HEPES, 298 K, *λ*_exc_ 345 nm).

Variations in the emission intensity and lifetime of the Eu and Tb complexes were examined systematically in the presence of dopamine, l-DOPA, HVA and each enantiomer of Trolox. In every case, Trolox proved to be the most effective quencher (Table 1). The values shown represent the mean values from three independent measurements (raw data are available *via* the SI). Trolox is arguably the most effective quenching species that has been identified that shortens the excited state lifetime of well-defined Eu or Tb complex by charge transfer. Its quenching behaviour fitted well to a classical Stern–Volmer analysis, with linear reductions of similar magnitude for emission lifetime and intensity, with both Eu and Tb complexes (Fig. 2), consistent with a dominant *dynamic* quenching mechanism. A small but significant difference was observed in quenching by the enantiomers of Trolox, with the *R* isomer, for example, quenching the racemic mixture of [TbL^1^]^+^ more strongly than the *S* enantiomer (Fig. 2).

**Table 1:** Stern–Volmer constants (*K*_SV_^−1^, μM) for dynamic quenching of the lanthanide excited state (pH 7.4, 0.1 M HEPES, 298 K). Values in parenthesis are estimates derived from emission intensity rather than lifetime variations

Quencher	[EuL^1^]^+^	[TbL^1^]^+^
Dopamine	404[112] (144[4])	36[0.5] (34[2])
DOPA^a^	65[3] (107[25])	8.2[0.2] (5.6[0.2])
Homovanillic acid	1299[21] (1078[63])	131[0.3] (162[0.6])
(*S*) Trolox	22[0.1] (20[0.1])	2.5[0.1] (2.9[0.1])
(*R*) Trolox	18[0.1] (17[0.2])	2.1[0.1] (2.7[0.1])

a The non-linear behaviour observed with [EuL^1^]^+^ renders the quenching constant estimate particularly imprecise, in this case. Values in square brackets represent the experimental errors from 3 independent analyses.

**Fig. 3:**
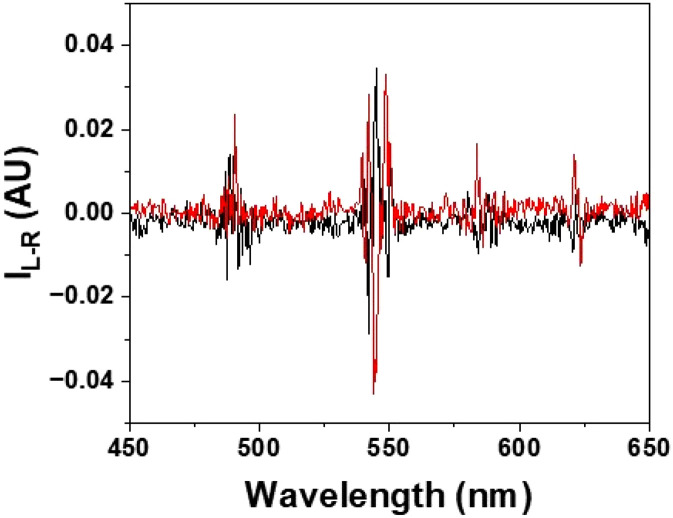
CPL spectra observed at steady state, following addition of *S* (black) or *R* (red) Trolox (15 μM) to [TbL^1^]^+^ (30 μM) (295 K, pH 7.4 HEPES 0.1 M, *λ*_exc_ 350 nm).

**Fig. 4:**
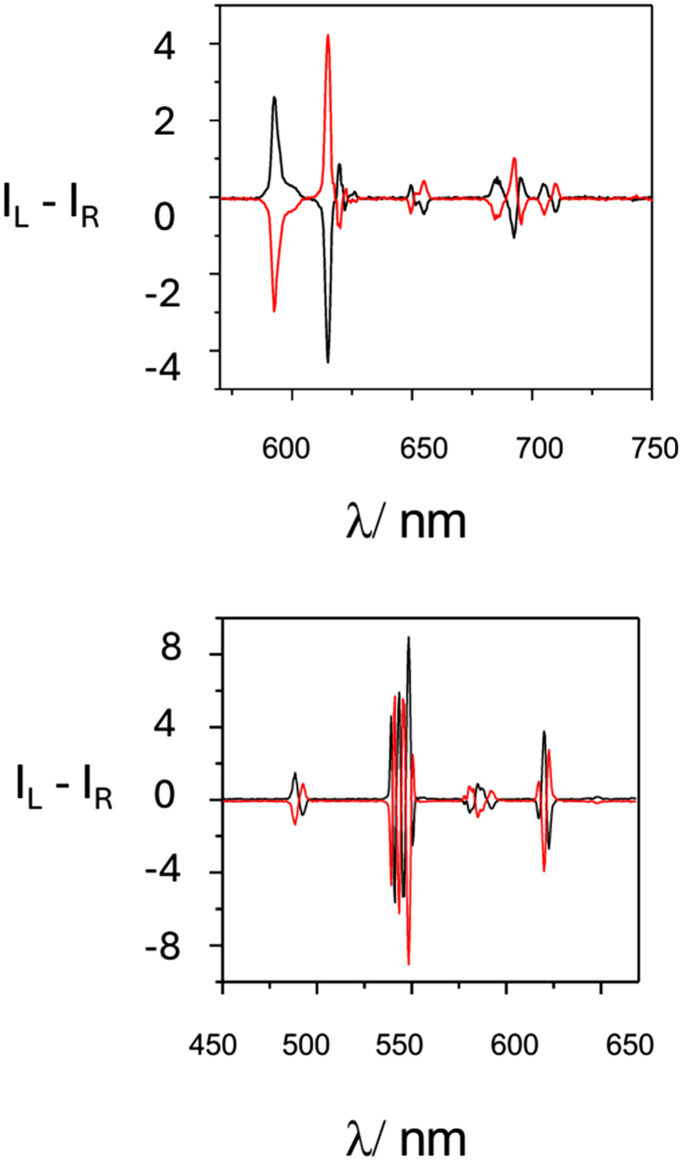
Upper: CPL spectra of *Δ* (black) and *Λ* (red) [EuL^4b^] (D_2_O, 295 K, *λ*_exc_ 350 nm); scaled by ×100 from the total emission spectrum; lower: *Δ* (black) and *Λ* [TbL^4b^] under the same conditions: note the sign of the strongest CPL signal in the Δ*J* = −1 and −2 bands at 485 and 548 nm.

The addition of *S*-Trolox reduces the total emission intensity and makes it difficult to gain sufficient signal intensity to record CPL spectra quickly. However, it gave rise to a weak but reproducible induced terbium CPL spectrum, and a slightly more intense CPL signal of opposite sign was observed when *R*-Trolox was added, notwithstanding the large reduction in total emission intensity that occurred almost equally for each enantiomer (Fig. 3). By comparison with the CPL signature of partly racemised complex, freshly isolated after chiral HPLC separation, with the chiroptical signatures of the *Δ* and *Λ* enantiomers of [LnL^4a^] and [LnL^4b^], structurally related analogues of established absolute configuration,^27–29^ (Fig. 4), it was deduced that *R*-Trolox gives rise to an excess of the *Λ* isomer, and *vice versa*.

Thus, *R*-Trolox quenches the *Δ* isomer more effectively, thereby defining an unusual new example of stereoselective (chiral) quenching of the terbium excited state. Such behaviour can only occur if the chiral, electron-rich Trolox aromatic ring and the electron poor dpq moiety are in close contact, during the millisecond lifetime of the lanthanide excited state.

**Fig. 5:**
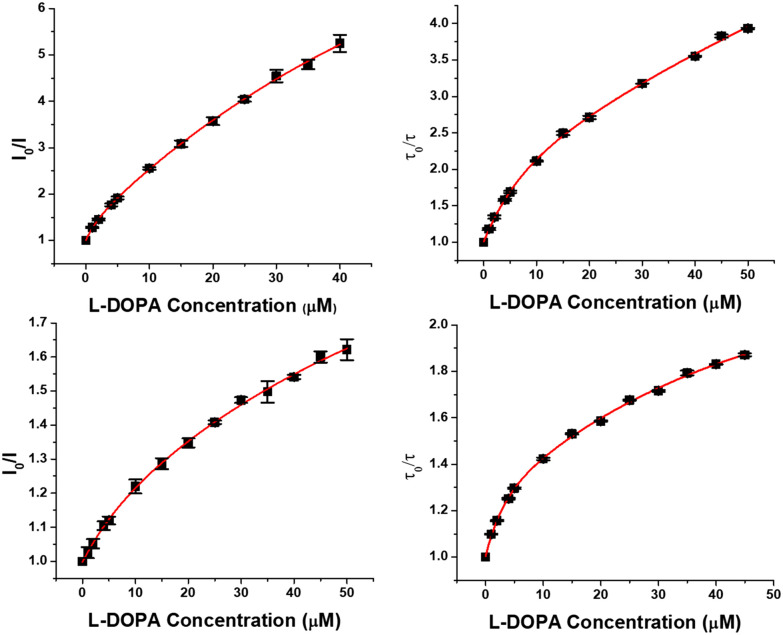
Variation of the [TbL^1^]^+^ emission intensity (upper left) or lifetime (upper right) *vs*. l-DOPA concentration; equivalent data for [EuL^1^]^+^ is shown below, (15 μM complex, pH 7.4, 0.1 M HEPES, 298 K). The pronounced non-linearity suggests the occurrence of an intermediate exciplex, as noted earlier with urate quenching.^22^

Amongst the three remaining quenching species examined, DOPA was most effective (Fig. 5), followed by dopamine (Fig. S3); HVA was the least efficient quencher by over an order of magnitude, and gave rise to almost perfectly linear Stern–Volmer plots, over the larger concentration range examined (Fig. S4), in contrast to the other two species. In every case, the Tb complex proved about ten times more effective than the Eu analogue, aligning with the 38 kJ mol^−1^ greater energy of the Tb ^5^D_4_ excited state (244 kJ mol^−1^), compared to the Eu ^5^D_0_ state.

Similar behaviour has been reported in the quenching of the lanthanide excited state in a wide range of Eu and Tb complexes by urate, (*E*_1/2_ +0.59 V, pH 7) ascorbate (+0.35 V) and iodide (+0.54 V). In that case, urate was the most efficient quenching species, notwithstanding its significantly higher one electron oxidation potential.^22,23,25^

Dynamic quenching requires collisional encounter and is normally both thermally activated and controlled by the local electrostatic gradient. It is evident that encounter in the cases studied here is determined neither by the charge nor the oxidation potential of the quenching species. The anion HVA was the least effective, (Table 1) and neutral l-DOPA was a more effective quencher than protonated dopamine at pH 7.4, in part at least because longer range approach of DOPA to the cationic complex is not inhibited by charge repulsion.

### Quenching mechanism: temperature dependence

The temperature dependence of [LnL^1^]^+^ quenching was examined to find out if there was any direct evidence for exciplex formation between the excited state of the electron poor tetrazatriphenylene moiety and the electron rich quenching species. Such a process brings two species together and is entropically unfavourable; higher temperatures can lead to reduced quenching and an increase in emission intensity, when there is a large unfavourable entropy contribution to the free energy of exciplex formation. In contrast, an increase in temperature may be expected to give rise to greater collisional quenching in a classical dynamic quenching process.

The emission intensity of [EuL^1^]^+^ decreased in the presence of added l-DOPA by 49% as the temperature increased from 25 to 45 °C. In parallel, the lifetime decreased by 41%. Near identical behaviour was found with [TbL^1^]^+^ and reductions of around 40% were observed in both lifetime and total emission intensity (Fig. S5). However, in the presence of *S* Trolox, the emission intensity and lifetime of [EuL^1^]^+^ decreased by only 8% and 4%, respectively, as temperature increased from 25 to 45 °C. Similar reductions were observed in quenching by *R* Trolox at higher temperatures (Fig. S6 and S7). For [TbL^1^]^+^, emission intensity and lifetime decreased by 26% and 22% with *S* Trolox, respectively, and similar changes were observed for *R* Trolox quenching. Notably, in assessing the Trolox temperature dependence, Eu quenching is almost temperature independent, which was not observed in l-DOPA quenching.

Taken together, such behaviour is characteristic of a predominant thermally activated quenching process, that is commonly determined by diffusion rates. A strong possibility is that transient formation of a quencher/chromophore π complex occurs in the excited state, lowering the energy of the dpq triplet. Higher temperatures lead to an increase in the rate of back energy transfer from the lanthanide excited state, repopulating the higher vibrational levels of the dpq triplet excited state, as noted in recent work.^11^ The Eu complex with Trolox showed very little temperature dependence for quenching in the range 25 to 45 °C, perhaps because the Eu acts as a charge sink, favouring the transfer of charge (arguably characterised by a larger Δ*H* term) from Trolox to the Eu-coordinated dpq chromophore in its triplet excited state. Indeed, the non-identical temperature dependence for Trolox quenching of the Eu and Tb complex excited states, supports this possibility.

A more detailed mechanistic understanding may come from future picosecond and nanosecond absorption spectroscopy experiments, monitoring the transient absorbance of the chromophore triplet excited state, in the absence and presence of the quenching species. Such experiments are to be undertaken in the near future and will be reported separately.

## Conclusions

Chirality is well known to be a key factor in molecular recognition in the excited state, but its influence in charge-transfer processes and especially in photoinduced bimolecular electron transfer remains controversial. The fine details of the mechanism are often simply considered and described in terms of the formation of diastereoisomeric excited state species of different free energy. Such a consideration is, of course, valid but does not reveal the true nature of chiral discrimination.^35,36^

Most reported studies have focused on examples of chiral fluorescence quenching, where the key role of diffusion has been highlighted with the short-lived excited states usually involved. With the lanthanide systems here, the much longer-lived metal excited state means there are many more diffusional encounters of the excited complex with the quenching species, so that time-averaged chiral discrimination can be assessed by steady-state measurements of lanthanide emission lifetime or intensity variation.

Much of the previous work on the ‘enantioselective quenching’ of lanthanide excited states examined the behaviour of racemic [Ln(DPA)_3_]^3−^ complexes (DPA is the tridentate ligand, dipicolinic acid). These tris-chelate complexes cannot be resolved at ambient temperature, as they racemise very quickly on the laboratory timescale. Several examples were identified that were hypothesised to involve stereoselective electronic energy transfer to the quenching species, although in some cases, changes in the constitution of the primary emissive species, under the reaction conditions, may have been overlooked.^19,37^

Here, we hypothesise that chiral discrimination must occur *via* the close encounter between the excited *Δ* and *Λ* enantiomers of the Tb and Eu complexes of L^1,2^ with *S* or *R* Trolox. This process can only occur in an exciplex. *R*-Trolox quenches the *Δ* isomer more effectively than the *Λ*, giving rise to a weak CPL signal characteristic of the *Λ* enantiomer, in accord with the relative quenching efficiencies found by following total emission and lifetime changes.

Surprisingly, DOPA was found to be a significantly more effective quencher than either dopamine or HVA with [TbL^1^]^+^ particularly, *via* a dynamic quenching process that was only slightly less efficient than with Trolox. Presumably this process also involves exciplex formation between the chromophore triplet excited state and the electron rich quenching species. The quenching sensitivity of these complexes, however, remains insufficient to allow quantitative analysis of l-DOPA in serum samples, as they contain urate at a rather high relative concentration (100 to 450 μM); urate dominates the quenching behaviour of these complexes, in such serum mixtures.

## Author contributions

The manuscript was written by DP with contributions from each author; XW carried out the syntheses and characterisation work and undertook the measurements of binding and rate constants at steady state; DJB and RP undertook CPL measurements at Durham University.

## Conflicts of interest

There are no conflicts to declare.

## Supplementary Material

DT-055-D5DT03023D-s001

## Data Availability

The additional experimental data associated with this article have been included in the supplementary information (SI). Supplementary information: analytical methods and photophysical data. See DOI: https://doi.org/10.1039/d5dt03023d. Any additional data such as sets of text files for variations of emission lifetime for Tb or Eu complexes and their temperature dependence (3 replicates in each case at several temperatures), additional emission spectra and lifetime variations as a function of added quencher, can be accessed by request to the corresponding author.
