# Supplementary material for: Chemoselective and stereoselective charge transfer dynamic quenching with triazacyclononane europium and terbium tetra-azatriphenylene complexes
Source: Dalton Trans. 2026 Mar 17;55(14):5602–7. doi: 10.1039/d5dt03023d (PMC13001716; doi:10.1039/d5dt03023d)
Supplement: DT-055-D5DT03023D-s001 [file DT-055-D5DT03023D-s001.pdf]

Supplementary Information (SI) for Dalton Transactions.
This journal is © The Royal Society of Chemistry 2026

Supplementary Information

Chemoselective and stereoselective charge transfer dynamic quenching with triazacyclononane europium and terbium tetra-azatriphenylene complexes

Xinyi Wen [a], Dominic J. Black [b], Robert Pal [b] and David Parker [a*†]

a) Department of Chemistry, Hong Kong Baptist University, Kowloon Tong, 999077, Hong Kong, China
b) Department of Chemistry, Durham University, South Road, Durham DH1 3LE, UK

1. General Procedures; HPLC, optical techniques. Pages 1-3
2. Synthetic Procedures Pages 4-8
3. HPLC traces of $[LnL^1]^+$ and $[EuL^2]^+$ Pages 9
4. CPL spectra of isolated enantiomers for $[TbL^1]^+$ and $[EuL^2]^+$ Pages 10
5. Quenching studies for $[LnL^1]^+$ with dopamine and HVA Pages 10-12
6. Temperature dependence studies for $[LnL^1]^+$ Pages 12-15
7. References Pages 15

EXPERIMENTAL

General procedures

All reagents were obtained from commercial sources and were used as received. Solvents were laboratory grade, and anhydrous solvents were obtained from a Solvent Purification System. Air sensitive and moisture sensitive reactions were conducted under an inert atmosphere of nitrogen using Schlenk-line techniques. Converting counterions of complex to more soluble chloride salts was carried out using a DOWEX® 1x4 50-100 mesh Cl ion exchange resin (Sigma-Aldrich®).

Preparative column chromatography was performed using a Biotage® Isolera™ instrument, equipped with a 200-800 nm UV-Vis detector and Biotage® Rening Cartridge – High Performance 40-63 µm columns. Crudes were loaded directly onto silica gel (Bidepharm, 100-200 mesh).

Thin-layer chromatography was performed on silica plates and visualised using a portable UV lamp (254/365 nm).

Electrospray mass spectra were recorded using a SCIEX 3200Q ESI mass spectrometer for monitoring reaction and determining collected fraction data, during purification of product. Methanol or acetonitrile was used as the carrier solvent. **Accurate masses** were obtained on a Bruker Autoflex MALDI-TOF mass spectrometer.

^{1}H and ^{13}C NMR spectra were obtained using a 9.4 T Bruker Ultrashield 400 Plus NMR spectrometer (^{1}H NMR at 400 MHz; ^{13}C NMR at 101 MHz) at 295 K. The ^{1}H NMR chemical shifts were referenced to the corresponding solvent peak (7.16 for $CDCl_3$). Deuterated solvents were all commercially available. All chemical shifts are given in ppm with coupling constants in Hz.

HPLC analysis

HPLC analyses and purifications were performed with Agilent system at 295 K. All chromatograms report the absorbance monitored at 254 nm.

Agilent system. Agilent 1100 module HPLC system (Agilent Technologies, Stockport, UK), G1313A Autosampler (Micro-WPS), G1312A Binary Pump, G1315A Diode-Array Detector (DAD) and Agilent 5 HC-C18 (2) column (5 µm, 4.6 x 250 mm).

- *Method A: (Agilent system)* flow rate 0.5 mL/min with H_2O (0.1% TFA) – 20% MeCN (0.1% TFA) as eluents (linear gradient to 80% MeCN (0.1% TFA) [40 min].

Shimadzu system. Semi-preparative High Performance Liquid Chromatograph LC-20AR, LC-20AR Solvent Delivery Pump, DGU-40 Degassing unit, LH-40 Liquid Handler, SPD-M40 Photodiode Array Detector, FRC-40 Fraction Collector, CBM-40 System Controller and XBridge® Prep C18 OBD™ column (5 µm, 19 × 100 mm).

- *Method B: (Shimadzu system)* flow rate 5.0 mL/min with H_2O (0.1% TFA) – 20% MeCN (0.1% TFA) as mobile phases (gradient to 47% MeCN (0.1% TFA) [27 min].
- *Method C: (Shimadzu system)* flow rate 5.0 mL/min with H_2O (0.1% TFA) – 30% MeCN (0.1% TFA) as mobile phases (gradient to 66% MeCN (0.1% TFA) [24 min].

Waters system. Waters 2707 Autosampler, Water 1525 Binary HPLC Pump, Waters 2998 Photodiode Array Detector and Waters Fraction Collector III and Atlantis® T3 Prep OBD™ C18 column (5 µm, 19 × 250 mm).

- *Method D: (Waters system)* flow rate 5.0 mL/min with H_2O (0.1% TFA) – 30% MeCN (0.1% TFA) as mobile phases (gradient to 60% MeCN (0.1% TFA) [24 min].
- *Method E: (Waters system)* flow rate 5.0 mL/min with H_2O (0.1% TFA) – 30% MeCN (0.1% TFA) as mobile phases (gradient to 66% MeCN (0.1% TFA) [34 min].

Chiral HPLC was performed on the *Shimadzu system* described above using an analytical CHIRALPAK-IB N-5 250 x 4.6 mm 5 µm column, with an isocratic solvent system of EtOH/MeOH/TEA/TFA (v/v/v/v 50:50:0.5:0.3).

Optical Techniques

UV/Vis absorbance spectra were recorded using an Agilent Technologies Cary 8454 UV-Vis spectrometer.

Emission spectra were recorded on a HORIBA Scientific Fluoromax-4 luminescence spectrofluorimeter.

Lifetime measurements were carried out using a HORIBA Fluorolog-3 spectrometer under the stated conditions.

Temperature dependent measurements were carried out using Peltier-controlled cuvette holder supplied with magnetic stirring using T-App software.

Quantum yields were performed based on the previous method and the use of two lanthanide complex standards, as reported [1].

CPL spectra of isolated enantiomers were recorded with a 10 mm quartz cuvette on a CPL-300 spectrophotometer supplied by the JASCO corporation employing multiple spectral accumulations. The instrument uses a scattering angle of 0° from the excitation of unpolarized, monochromated incident light with a bandwidth of 30 nm and a bandwidth for emission of 30 nm. The scanning speed was 50 nm/min and the time constant PMT (photomultiplier tube) was 8 s.

CPL spectra of $[TbL^1]^+$ with Trolox addition were recorded with a home-built (modular) spectrometer. The excitation source was a broad band (200 – 1000 nm) laser- driven light source EQ 99 (Elliot Scientific). The excitation wavelength was selected by feeding the broadband light into an Acton SP-2155 monochromator (Princeton Instruments); the collimated light was focused into the sample cell (1 cm quartz cuvette). Sample PL emission was collected perpendicular to the excitation direction with a lens (f = 150 mm). The detection of the CPL signal was achieved using the field modulation lock-in technique. The electronic signal from the PMT was fed into a lock-in amplifier (Hinds Instruments Signaloc Model 2100). The reference signal for the lock-in detection was provided by the PEM control unit. The monchromators, PEM control unit and lock-in amplifier were interfaced to a desktop PC and controlled by a custom-written Labview graphic user interface. Spectral calibration of the scanning monochromator was performed using a Hg-Ar calibration lamp (Ocean Optics). The emission spectra were recorded with 0.5 nm step size, and the slits of the detection monochromator were set to a slit width corresponding to a spectral resolution of 0.25 nm. CPL spectra (as well as total emission spectra) were obtained through an averaging procedure of several scans. The CPL spectra were smoothed using a shape-preserving Savitzky-Golay smoothing (polynomial order 5, window size 9 with reflection at the boundaries) to reduce the influence of noise and enhance visual appearance; all calculations were carried out using raw spectral data. Analysis of smoothed vs raw data was used to help to estimate the uncertainty in the stated g_{em} factors, which was typically ±10%.

Synthetic Procedures

Ligands L1-2 (9-N-3-dpq) (Scheme 1)

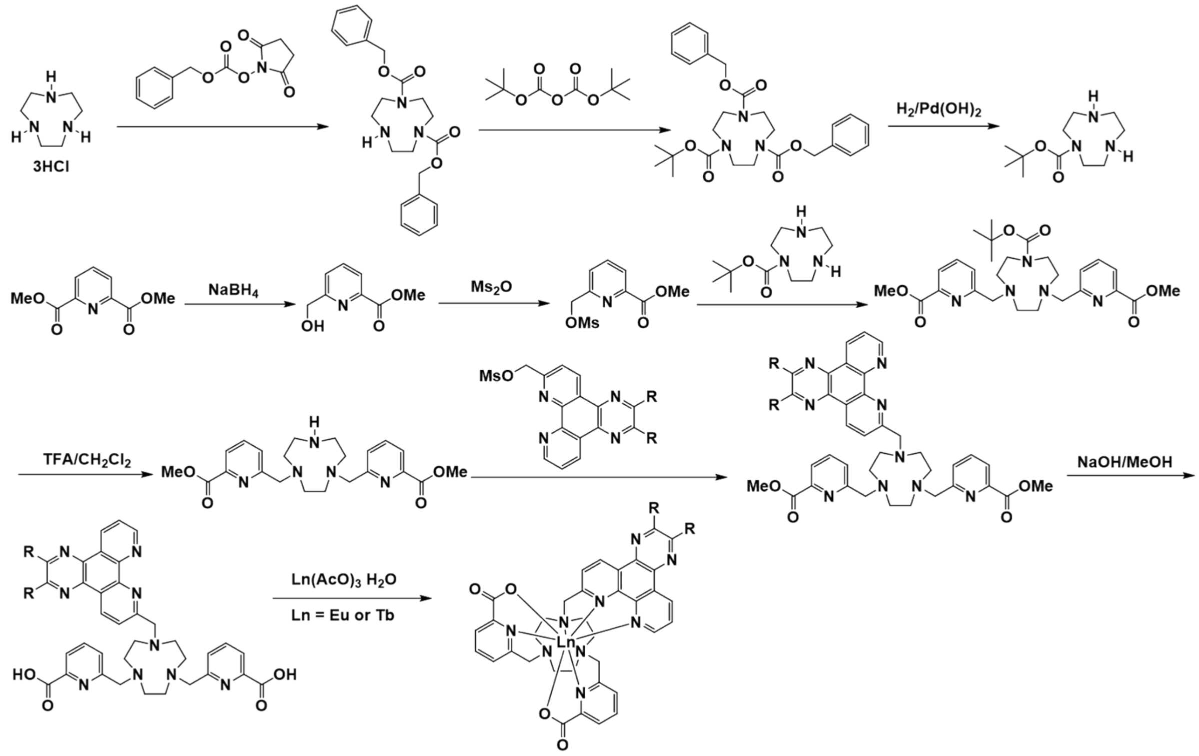

R = Me, L1

R = Ph, L2

L1 and L2 (as dimethyl esters) [2]

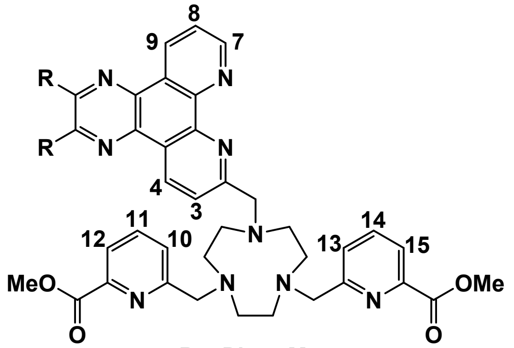

R = Ph or Me

The synthetic procedures for ligands L^1 and L^2 used minor modifications to the published work. [2]

In each case, the crude residue was purified by RP-HPLC (*Method B*, ***t***$_R$: 14.7 min for L^1; *Method C*, ***t***$_R$: 16.4 min for L^2) to afford the title complex as a dark yellow viscous oil. (**L^1**: 5 mg, 16 %; **L^2**: 7 mg, 20 %).

L^1: ^{1}H NMR (298 K, 400 MHz, $CDCl_3$) 10.02 (1H, d, $^3J_{H-H}$ 8.0 Hz, H^7), 9.58 (1H, d, $^3J_{H-H}$ 8.5 Hz, H^4), 9.48 (1H, d, $^3J_{H-H}$ 4.5 Hz, H^9), 8.23 (1H, m, H^8), 8.13 (1H, d, $^3J_{H-H}$ 8.5 Hz, H^3), 7.87 – 7.78 (4H, m, H^{11,12,14,15}), 7.52 (2H, d, $^3J_{H-H}$ 8.0 Hz, H^{10,13}), 4.85 (2H, s, CH_2Ar), 4.54 (4H, s, CH_2-py), 3.96 – 3.73 (8H, m, ring CH_2), 3.66 (6H, s, OCH_3), 3.58 (4H, s, ring CH_2), 2.89 (6H, s, CH_3); **^{13}C NMR** (298 K, 101 MHz, $CDCl_3$) δ 165.0 (2C, C=O), 156.4 (2C, q Ar), 155.5 (1C, q Ar), 155.5 (2C, q Ar), 146.8 (2C, q Ar), 146.1 (1C, C^9), 140.3 (1C, C^7), 138.3 (2C, C^{11,14}), 137.3 (1C, q Ar), 135.5 (1C, q Ar), 135.3 (1C, C^4), 129.3 (1C, q Ar), 128.4 (1C, q Ar), 127.3 (1C, C^3), 126.6 (2C, C^{10,13}), 125.3 (1C, C^8), 124.4 (2C, C^{12,15}), 61.7 (1C, CH_2Ar), 61.3 (1C, CH_2-py), 54.7 (2C, ring NCH), 54.2 (2C, ring NCH), 53.0 (2C, ring NCH), 52.8 (2C, OCH_3), 23.2 (2C, CH_3); **ESI-MS** (+) *m/z* 699.989 [M+H]$^+$; **ESI-HRMS (+)** calc. $[C_{39}H_{42}N_9O_4]^+$ 700.3354, found 700.3375.

^{1}H NMR spectrum ($CDCl_3$, 400 MHz, 298 K) of L^1

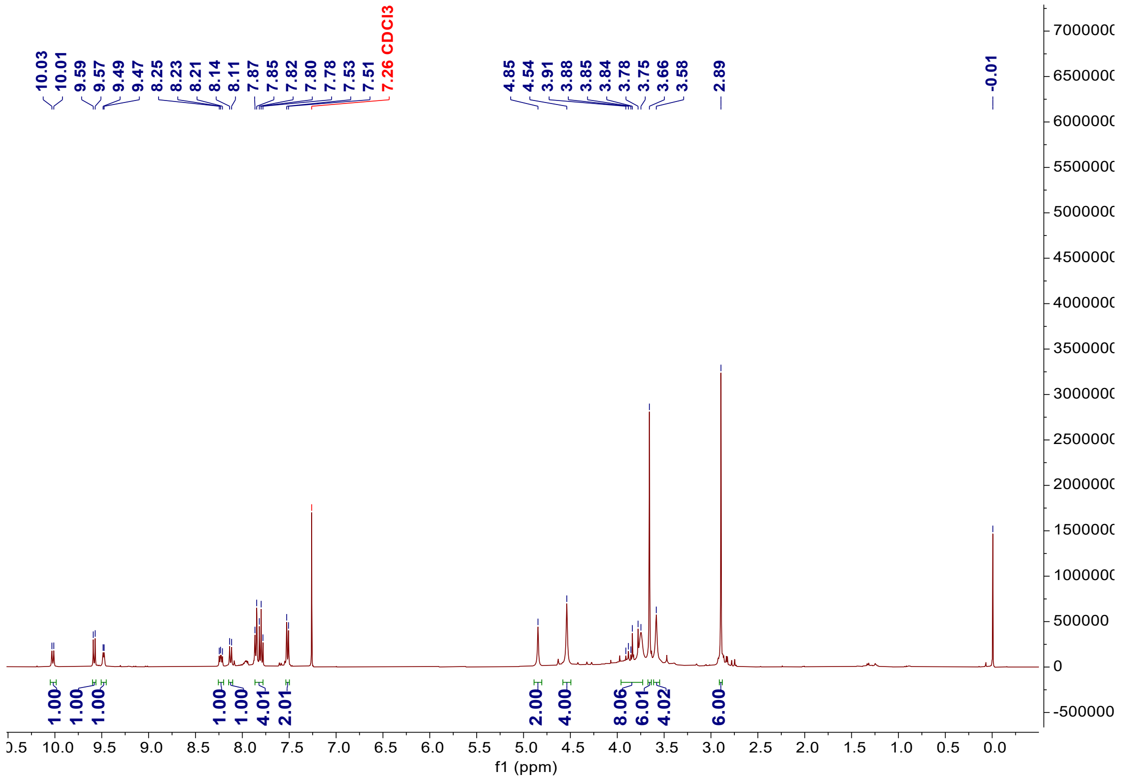

^{13}C NMR spectrum ($CDCl_3$, 101 MHz, 298 K) of L^1

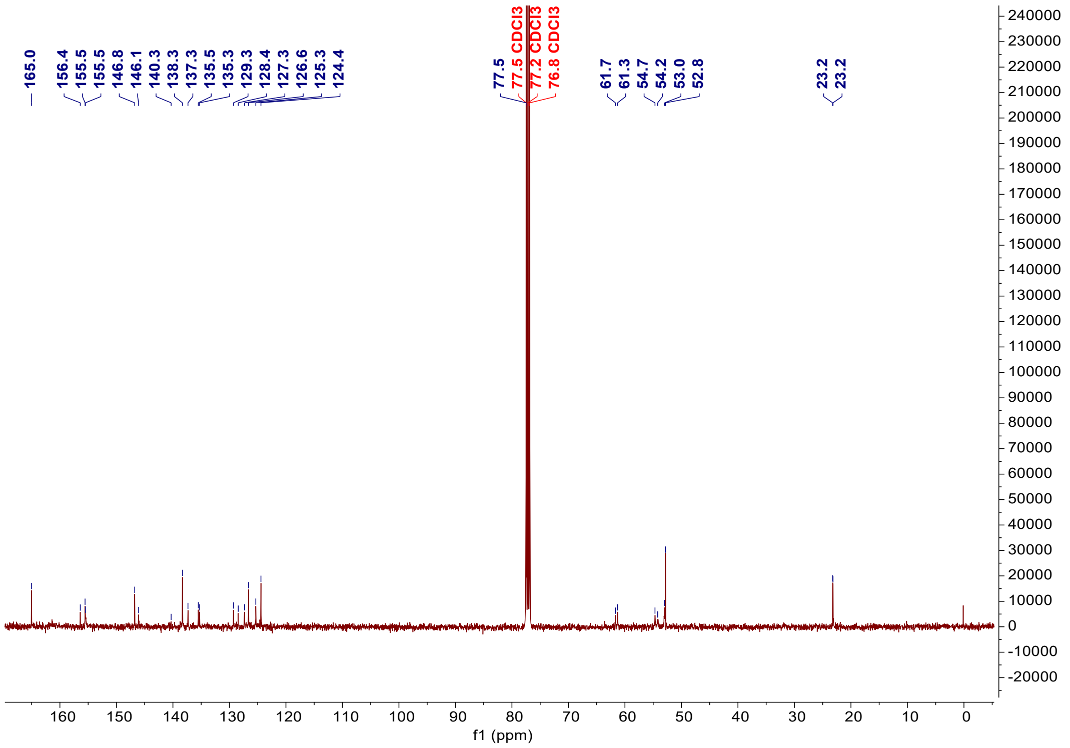

L^2: ^{1}H NMR (298 K, 400 MHz, $CDCl_3$) 10.26 (1H, d, $^3J_{H\text{-}H}$ = 8.0 Hz, H^7), 9.78-9.69 (1H, m, H^4), 9.51 (1H, br, H^9), 8.42 (1H, d, $^3J_{H\text{-}H}$ = 8.5 Hz, H^8), 8.21 (1H, br, H^3), 7.81 (4H, m, $H^{11,12,14,15}$), 7.72 (4H, m, $H^{10,13}$ and Ar-H), 7.51-7.41 (8H, m, Ar-H), 5.08 (2H, s, CH_2Ar), 4.63 (4H, s, CH_2-py), 4.11-3.72 (12H, m, ring CH_2), 3.55 (6H, s, OCH_3); **^{13}C NMR** (298 K, 101 MHz, $CDCl_3$) δ 164.9 (2C, C=O), 155.8 (2C, q Ar), 155.0 (1C, q Ar), 153.5 (2C, q Ar), 146.3 (2C, q Ar), 145.3 (1C, C^9), 142.9 (1C, C^7), 139.8 (1C, q Ar), 138.8 (1C, q Ar), 138.5 (2C, $C^{11,14}$), 137.8 (1C, q Ar), 137.6 (1C, q Ar), 136.5 (1C, C^4), 135.2 (1C, q Ar), 130.3 (2C, Ar C), 130.2 (2C, Ar C), 130.1 (2C, Ar C), 129.8 (1C, q Ar), 129.1 (1C, q Ar), 128.8 (2C, Ar C), 128.7 (2C, Ar C), 128.2 (1C, C^3), 126.1 (2C, $C^{10,13}$), 126.1 (1C, C^8), 124.4 (2C, $C^{12,15}$), 61.6 (3C, $\underline{C}H_2$), 55.1 (2C, ring NCH), 53.6 (2C, ring NCH), 53.5 (2C, ring NCH), 52.8 (2C, OCH_3); **ESI-MS** (+) *m/z* 824.496 $[M+H]^+$; **ESI-HRMS (+) calc.** $[C_{49}H_{46}N_9O_4]^+$ 824.3667, found 824.3617.

^1H NMR spectrum ($CDCl_3$, 400 MHz, 298 K) of L^2

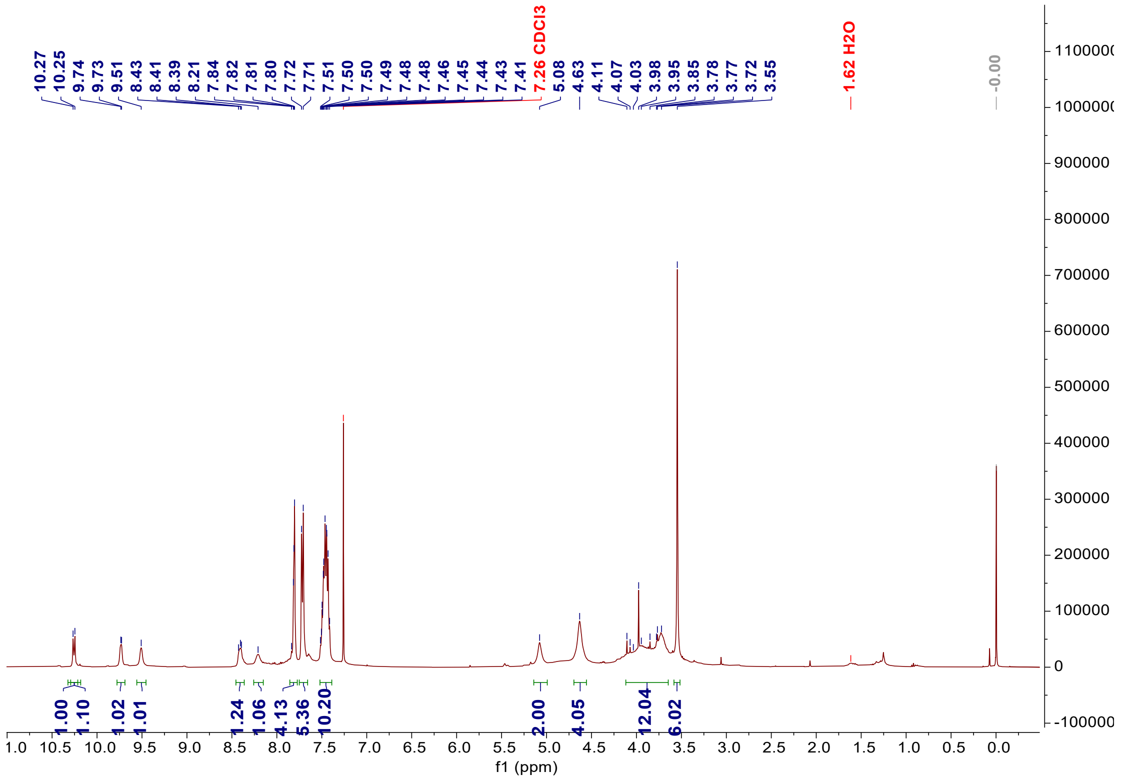

^{13}C NMR spectrum ($CDCl_3$, 101 MHz, 298 K) of L^2

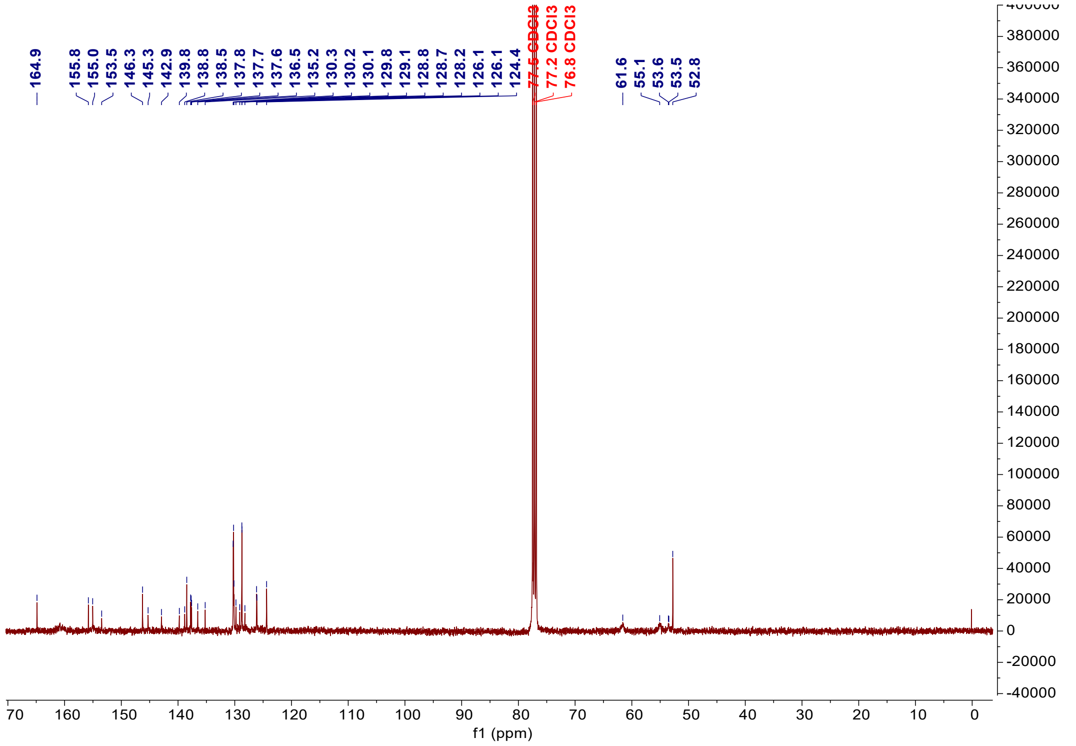

[LnL$^{1-2}$], (R = Me, L^1; R = Ph, L^2) (Ln = Eu or Tb)

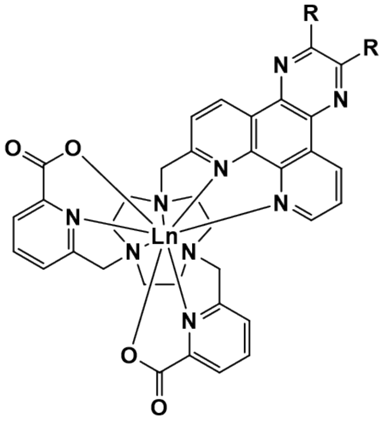

R = Ph or Me,
Ln = Eu or Tb

The dimethyl ester of L^1 or L ^{2} (0.012 mmol) was suspended in aqueous sodium hydroxide solution (5 mL, 0.1 M) and acetonitrile (ca. 2 mL) was added dropwise until the solid dissolved. The solution was stirred at room temperature for 16 h. The solution was neutralised using dilute hydrochloric acid and the solvent was removed under reduced pressure. The resulting solid was re-dissolved in acetonitrile (3 ml) and $Eu(OAc)_3$ or $Tb(OAc)_3$ (0.0144 mmol) was added. The reaction solution was stirred at 65 ˚C for 18 h. After this time, the solvent was removed under reduced pressure. The residue was purified by preparative HPLC (**LnL^1**: *Method D*, t_R: 15.4 min; **LnL^2**: *Method E*, t_R: 29.4 min)

[EuL^1]: ESI-HRMS (+) calc. $[C_{37}H_{35}EuN_9O_4]^+$ 822.2024, found 822.2025. τ_{H2O} (Eu) 0.96 ± 0.01 ms, λ_{max} 345 nm (3300 M^{-1} cm^{-1}). **[TbL^1]: ESI-HRMS** (+) calc. $[C_{37}H_{35}TbN_9O_4]^+$ 828.2065, found 828.2062. τ_{H2O} (Tb) 1.60 ± 0.01 ms, λ_{max} 345 nm (6000 M^{-1} cm^{-1}). **[EuL^2]: ESI-HRMS** (+) calc. $[C_{47}H_{39}EuN_9O_4]^+$ 946.2337, found 946.2323. τ_{H2O} (Eu) 0.95 ± 0.01 ms, λ_{max} 363 nm (30,000 M^{-1} cm^{-1}).

HPLC traces of [LnL^1]$^+$ and [EuL^2]$^+$ (*Method A*)

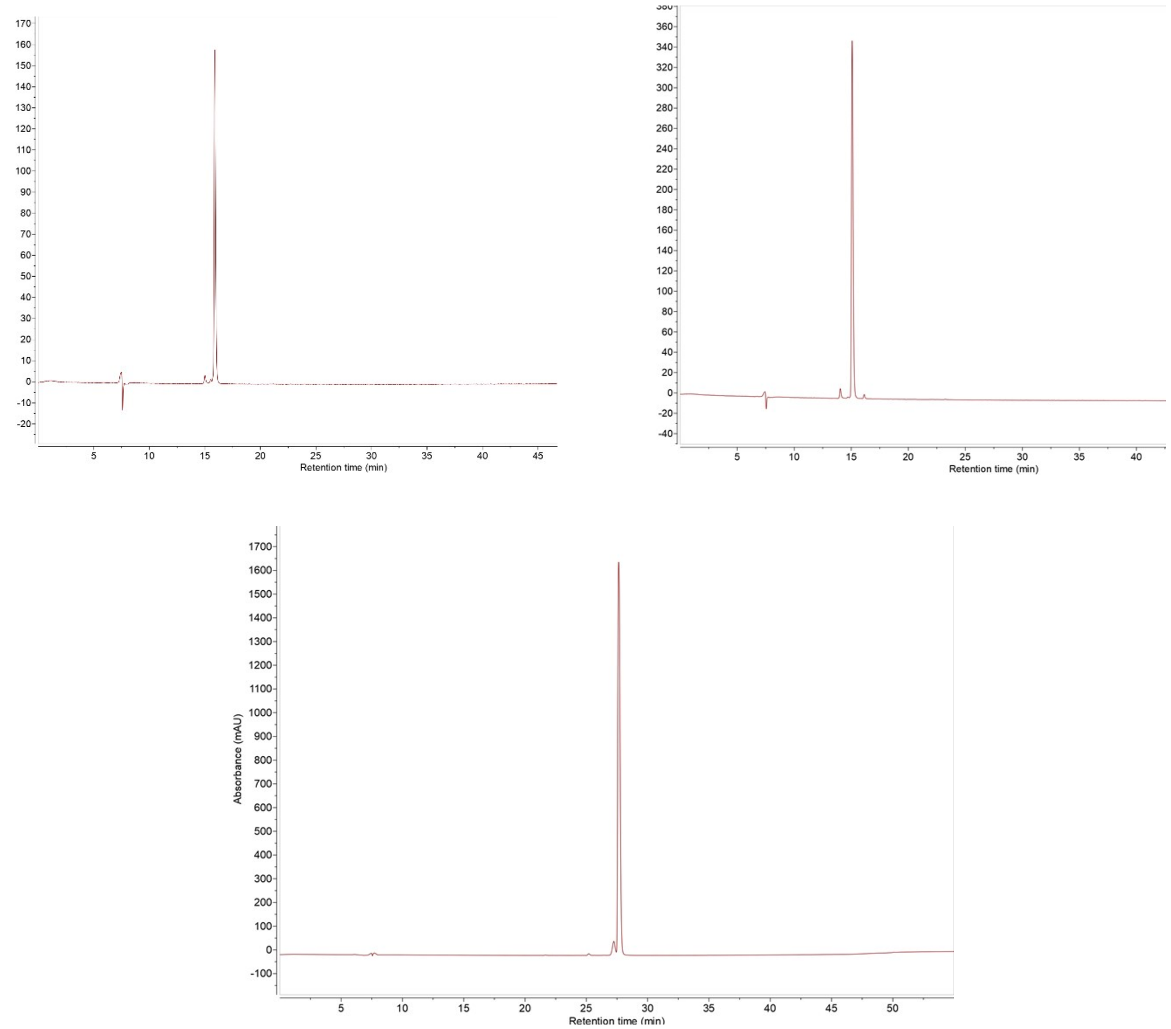

Fig. S1 HPLC traces of $[TbL^1]^+$ (*upper left*), $[EuL^1]^+$ (*upper right*) and $[EuL^2]^+$ (*lower*).

CPL spectra of isolated enantiomers for $[TbL^1]^+$ and $[EuL^2]^+$

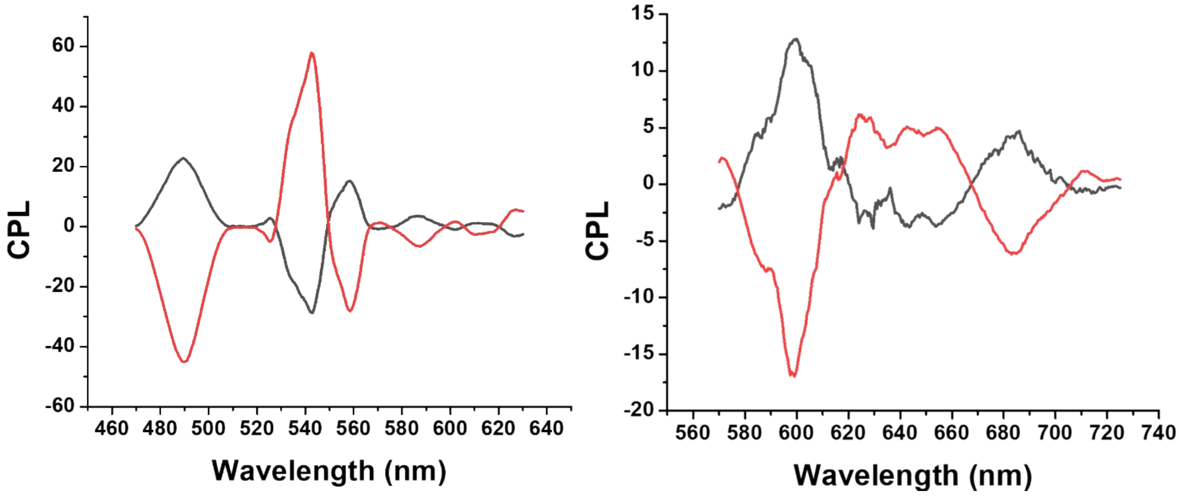

Fig. S2 CPL spectra of enantiomerically enriched samples of differing enantiomertic purity for $[TbL^1]^+$ (*left)* and $[EuL^2]^+$ (*right*), highlighting the different signs of the major transitions (298 K, D_2O, λ_{exc} 345nm for $[TbL^1]^+$ and λ_{exc} 364nm for $[EuL^2]^+$).

Quenching studies for $[LnL^1]^+$ with Dopamine and HVA

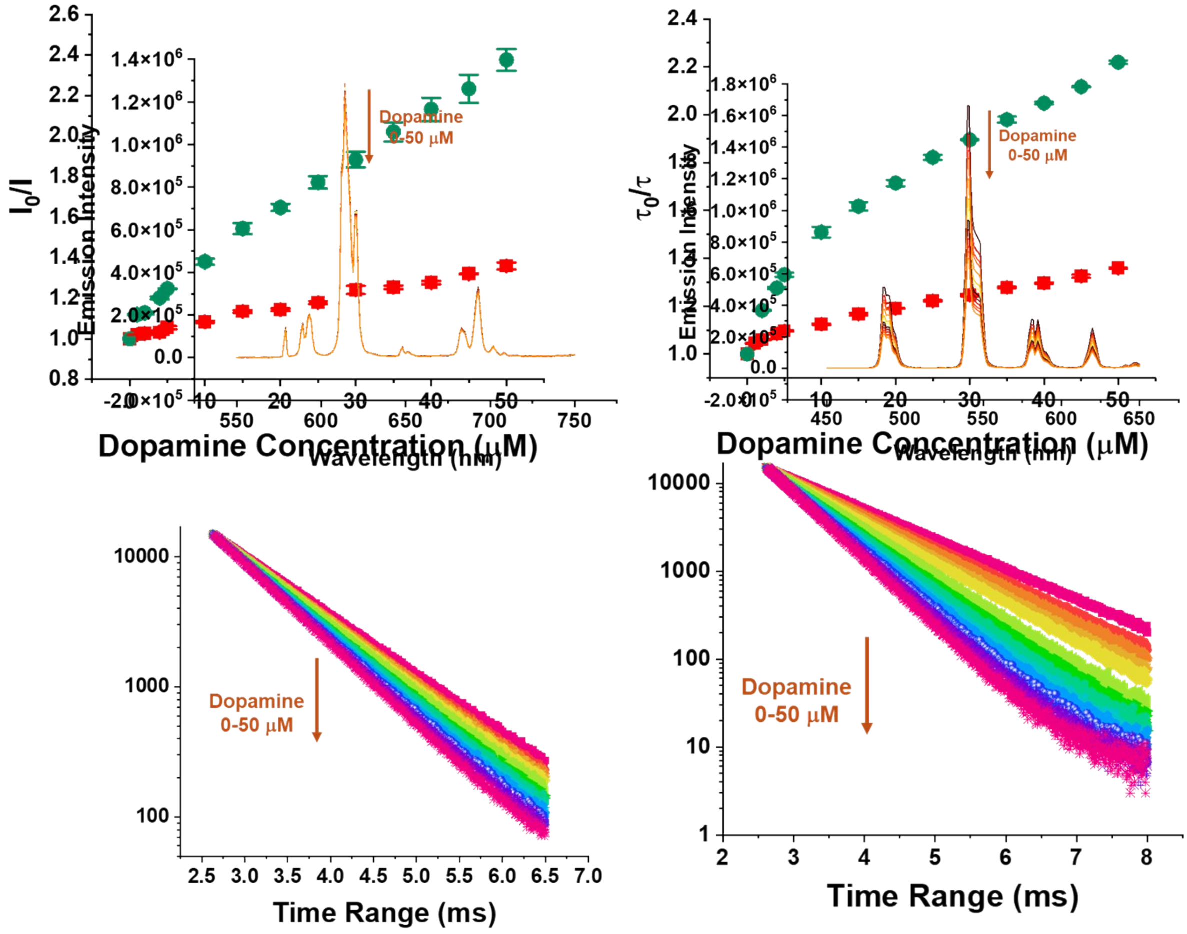

Fig. S3 Emission spectra (*Upper*) and lifetime spectra (*Middle*) of the $[LnL^1]^+$ (Ln = Eu, *left*; Ln = Tb, *right*) in the presence of different concentrations of the dopamine; *Lower*: variation of the $[LnL^1]^+$ (Ln = Eu, red square; Ln = Tb, green circle) emission intensities (*Left*) or lifetimes (*Right*) versus dopamine concentration. (I_0 = intensity without quencher, τ_0 = lifetime without quencher, 15 μM complex, pH 7.4, 0.1 M HEPES, 298 K).

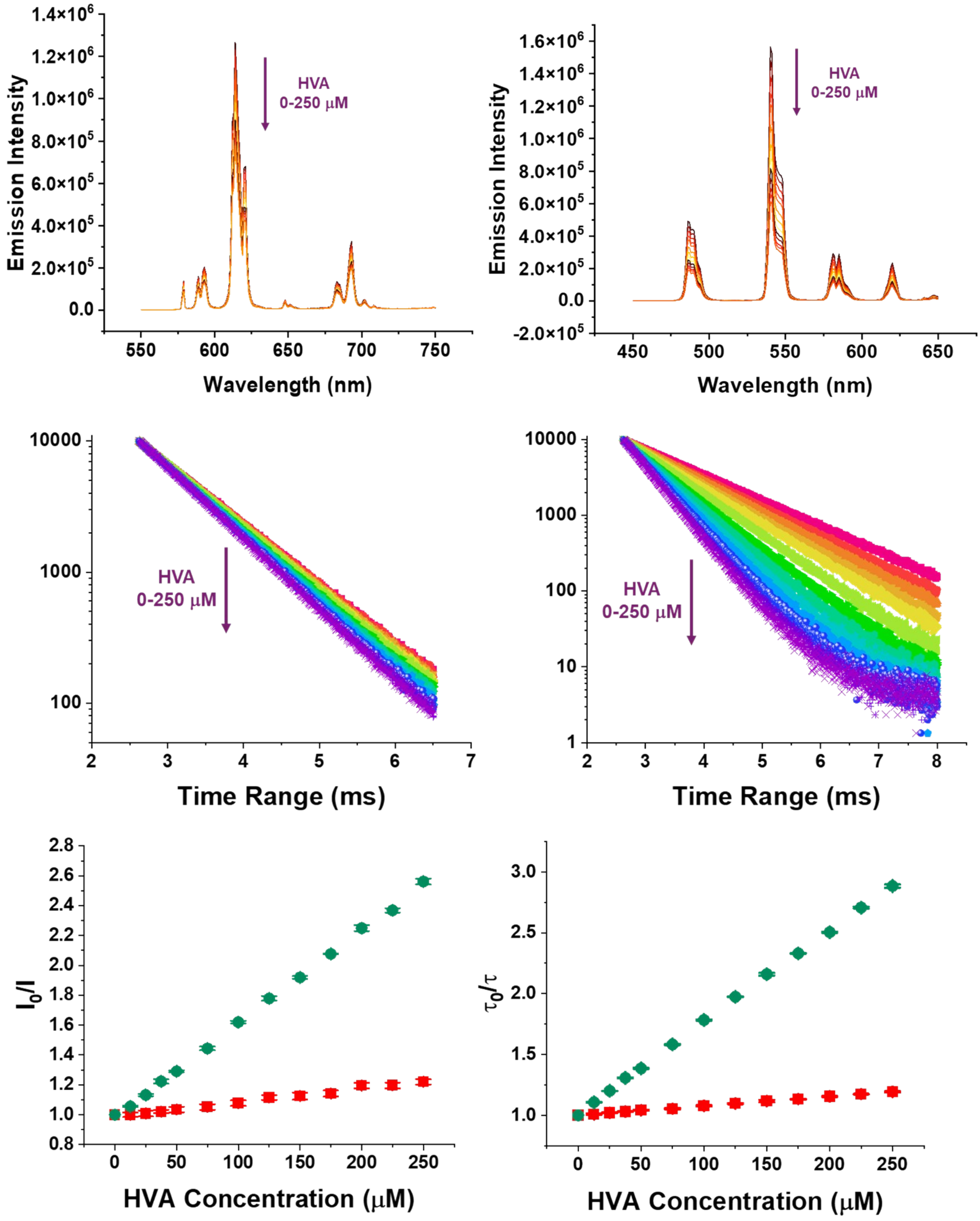

Fig. S4 Emission spectra (*Upper*) and lifetime spectra (*Middle*) of the $[LnL^1]^+$ (Ln = Eu, *left*; Ln = Tb, *right*) in the presence of different concentrations of the HVA; *Lower*: variation of the $[LnL^1]^+$ (Ln = Eu, red square; Ln = Tb, green circle) emission intensities (*Left*) or lifetimes (*Right*) versus HVA concentration. (I_0 = intensity without quencher, τ_0 = lifetime without quencher, 15 μM complex, pH 7.4, 0.1 M HEPES, 298 K).

Temperature dependence studies for $[LnL^1]^+$

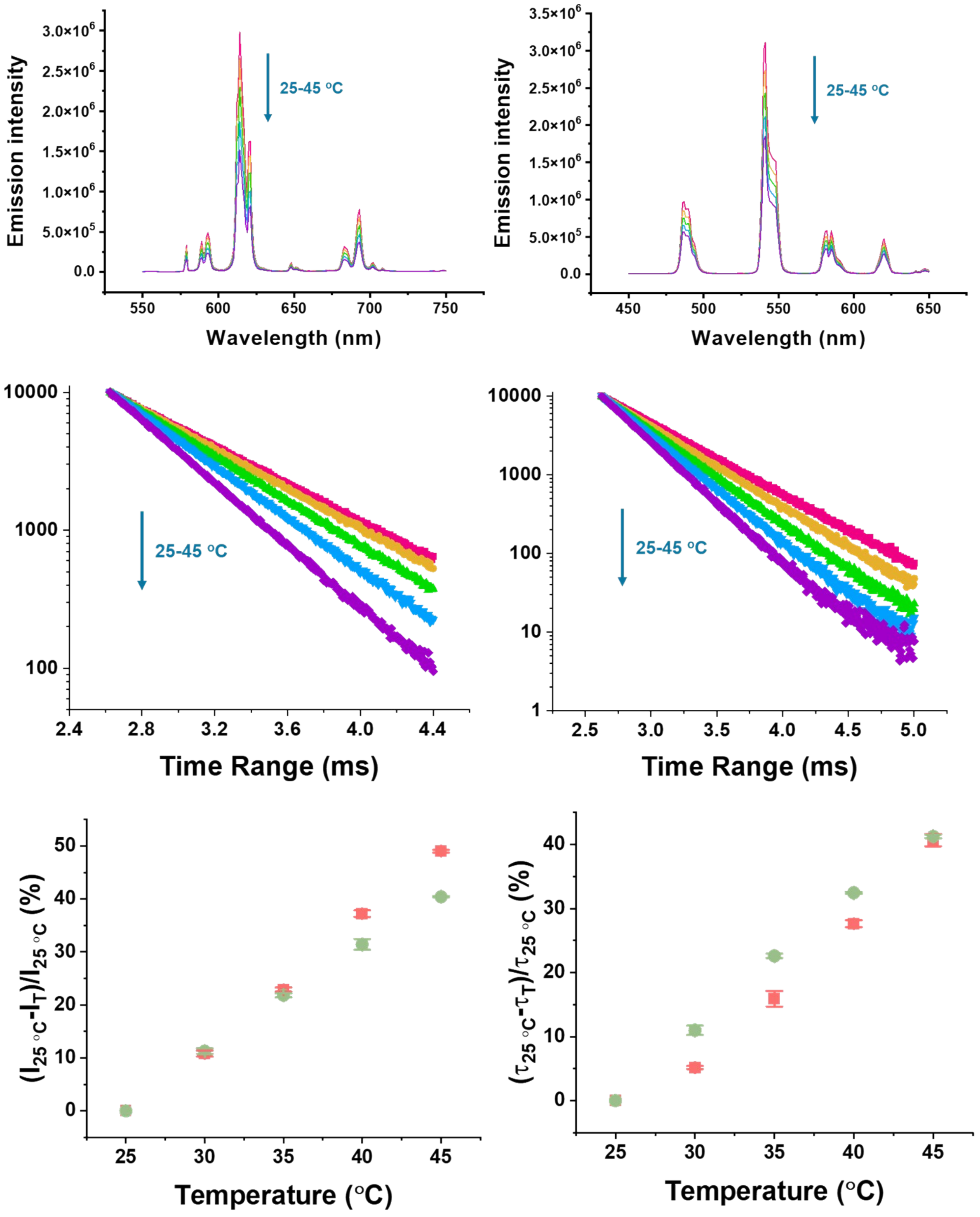

Fig. S5 Emission spectra (*Upper*) and lifetime spectra (*Middle*) of the $[LnL^1]^+$ (Ln = Eu, *left*; Ln = Tb, *right*) in the presence of the fixed concentration of the L-DOPA (65 μM for $[EuL^1]^+$, 10 μM for $[TbL^1]^+$); *Lower*: variation of emission intensities (*Left*) or lifetimes (*Right*) as a function of temperature for $[LnL^1]^+$ (Ln = Eu, red square; Ln = Tb, green circle) in the presence of L-DOPA. (15 μM complex, pH 7.4, 0.1 M HEPES, 298 K).

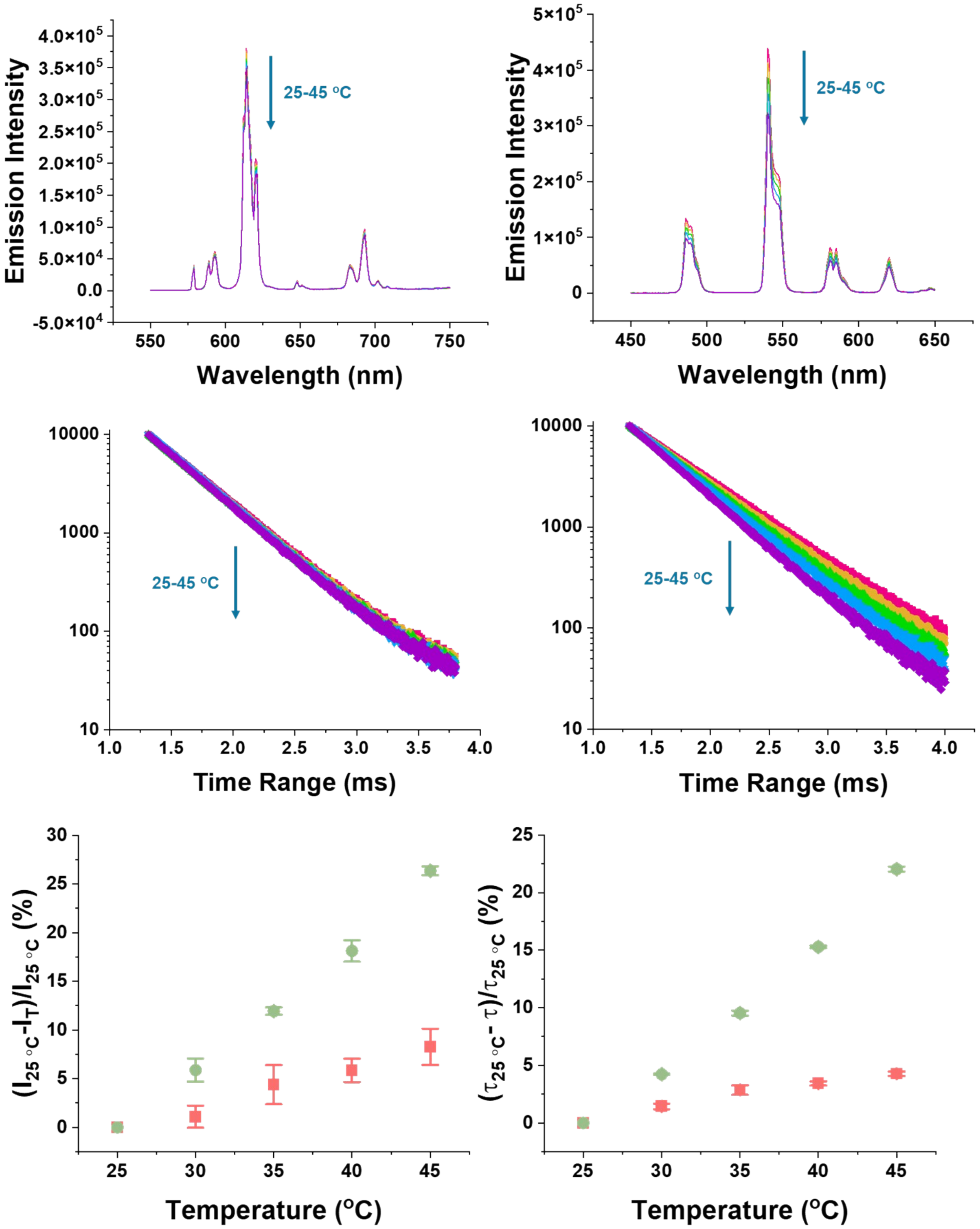

Fig. S6 Emission spectra (*Upper*) and lifetime spectra (*Middle*) of the [LnL^1]$^+$ (Ln = Eu, *left*; Ln = Tb, *right*) in the presence of the fixed concentration of the *S*-Trolox (20 μM for [EuL^1]$^+$, 3 μM for [TbL^1]$^+$); *Lower*: variation of emission intensities (*Left*) or lifetimes (*Right*) as a function of temperature for [LnL^1]$^+$ (Ln = Eu, red square; Ln = Tb, green circle) in the presence of *S*-Trolox. (5 μM complex, pH 7.4, 0.1 M HEPES, 298 K).

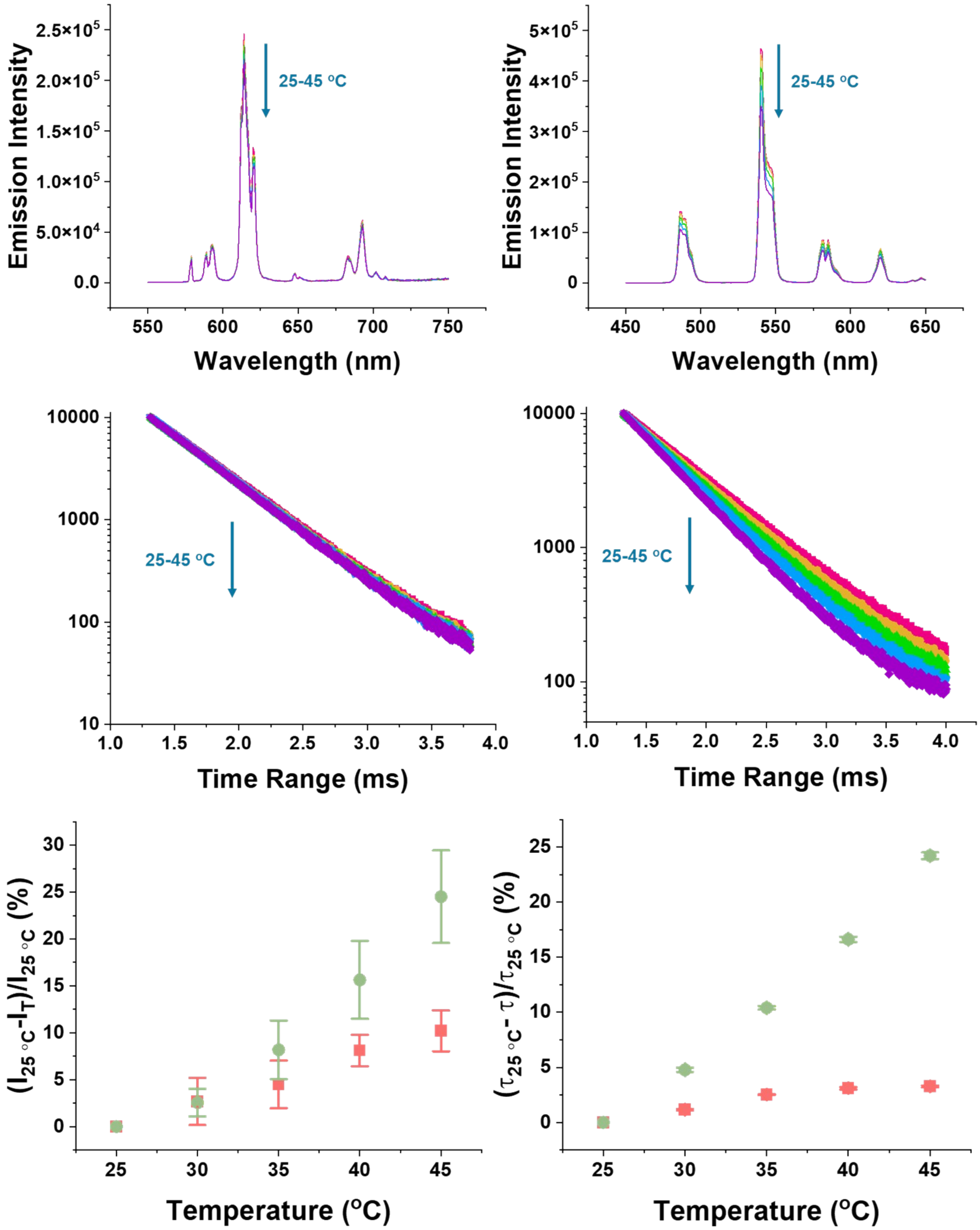

Fig. S7 Emission spectra (*Upper*) and lifetime spectra (*Middle*) of the $[LnL^1]^+$ (Ln = Eu, *left*; Ln = Tb, *right*) in the presence of the fixed concentration of the *R*-Trolox (17 μM for $[EuL^1]^+$, 2.5 μM for $[TbL^1]^+$); *Lower*: variation of emission intensities (*Left*) or lifetimes (*Right*) as a function of temperature for $[LnL^1]^+$ (Ln = Eu, red square; Ln = Tb, green circle) in the presence of *R*-Trolox. (5 μM complex, pH 7.4, 0.1 M HEPES, 298 K).

Reference

1. A. D'Aléo, A. Picot, A. Beeby, J. A. Gareth Williams, B. Le Guennic, C. Andraud and O. Maury, *Inorg. Chem.*, **2008**, 47, 10258–10268.
2. X. Y. Wen, H. S. Li, Z.J. Ju, R. R. Deng and D. Parker, *Chem. Sci.*, **2024**, 15, 19944-19951.
